# Editorial Note: Endoplasmic reticulum stress-induced autophagy provides cytoprotection from chemical hypoxia and oxidant injury and ameliorates renal ischemia-reperfusion injury

**DOI:** 10.1371/journal.pone.0346190

**Published:** 2026-04-02

**Authors:** The PLOS One Editors

Following the publication of this article [1], concerns were raised regarding the methods and results presented in Figs 1, 3, 7, and S1. Specifically,

- In Fig 3A, the β-Actin bands for the Cont and Scr-siRNA samples appear to be similar between the left and right β-Actin panels.
- In Fig S1A, the 24 Hour 3MA dot plot and histogram appear similar to the 24 Hour Tun +3MA dot plot and histogram.
- The Materials and Methods section does not report the number of animals used or the methods of anesthesia and euthanasia used in the study.

Regarding the β-Actin bands in Fig 3A, first author BBC stated that the same blot was re-probed with antibodies against Beclin1 and β-Actin after initially probing with ATG5, and that Fig 3A β-Actin left and right-side panels are the same image for the Con and Scr siRNA conditions. They provided the underlying images for these panels (S1 File).

First author BBC stated that the similarities between the 24 Hour 3MA and 24 Hour Tun + 3MA panels in Fig S1A likely arose due to errors during figure preparation, and that the underlying images for this figure are no longer available. They provided repeat data conducted before the time of the experiments in [1] for the 24 Hour 3MA and 24 Hour Tun + 3MA conditions (S2 File). PLOS does not consider the above concern for Fig S1A can be fully resolved without the original underlying data.

In regard to the animal experiments, first author BBC stated that a total of 105 mice were used in this study, with 24 mice used in Fig 6, 51 mice in Fig 7, and 30 mice in Fig 8. They stated that animals were anesthetized with 1–5% isoflurane and euthanized using gradual CO_2_ exposure.

The *PLOS One* Editors issue this Editorial Note to notify readers of the above clarification regarding Fig 3A, the details of the animal experiments, and the unresolved concerns regarding Fig S1A.

The corresponding author is deceased. Correspondence regarding this article may be sent to the first author, BBC (bhavyabc@gmail.com).

In addition to the concerns above, the *PLOS One* Editors are aware of similarities between results presented in Figs 1 and 7 of this article [1] and results presented in two articles published at a later date by different authors:

- The western blot panels of Figs 1C and 1D in this article [1] appear similar to Figs 1B and 1A, respectively, of [2, retracted in 3]. Partial underlying image data for these figs have been included as S3 File.
- Multiple panels within Fig 7C in this article [1] appear similar to panels in Fig 4 of [4].

## Supporting information

S1 File Original, uncropped images of the western blots presented in Fig 3A. (PDF)

S2 File Repeat data from before the time of the experiments in [1] for the 24 Hour 3MA and 24 Hour Tun + 3MA conditions in Fig S1A. Note: the FACS plots are gated differently to those in Fig S1 in [1].
(PDF)

S3 File Underlying image data for the LC3-I and LC3-II western blots presented in Figs 1C and 1D. (PDF)
