# Supplementary figures and images for: Editorial Note: Endoplasmic reticulum stress-induced autophagy provides cytoprotection from chemical hypoxia and oxidant injury and ameliorates renal ischemia-reperfusion injury
Source: PLoS One. 2026 Apr 2;21(4):e0346190. doi: 10.1371/journal.pone.0346190 (PMC13046123; doi:10.1371/journal.pone.0346190)

3A

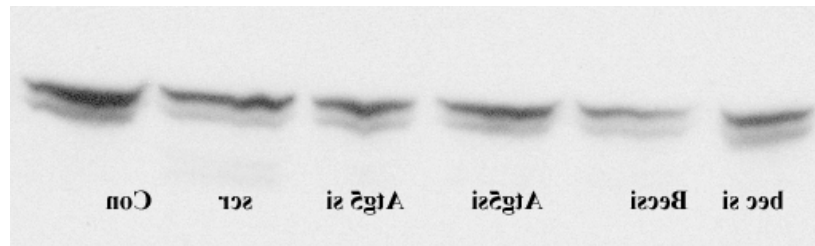

BECLIN

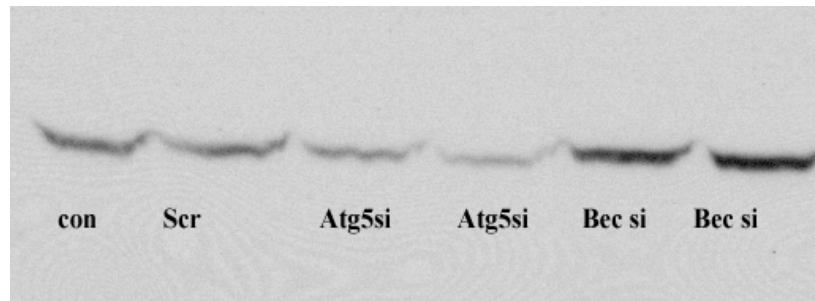

ATG-5

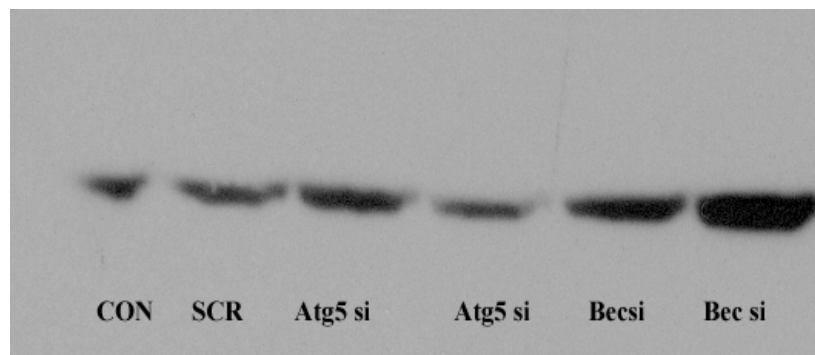

Beta ACTIN

Supplement: S1 File — (PDF) [file pone.0346190.s001.pdf]

# EXPERIMENT #1

3MA Alone-24h

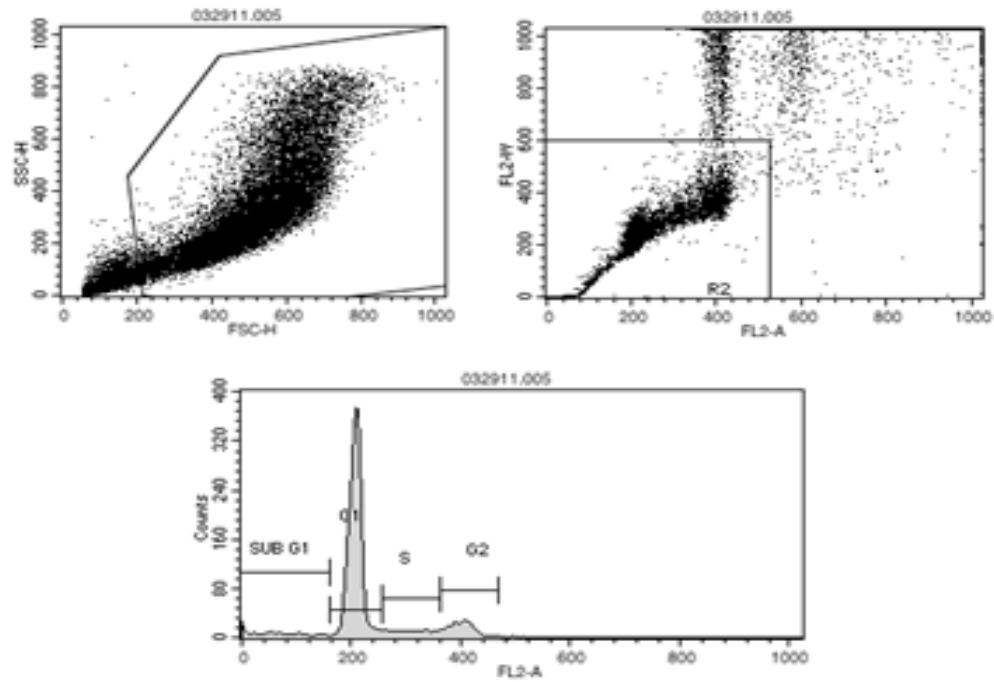

Tuni+3MA-24h

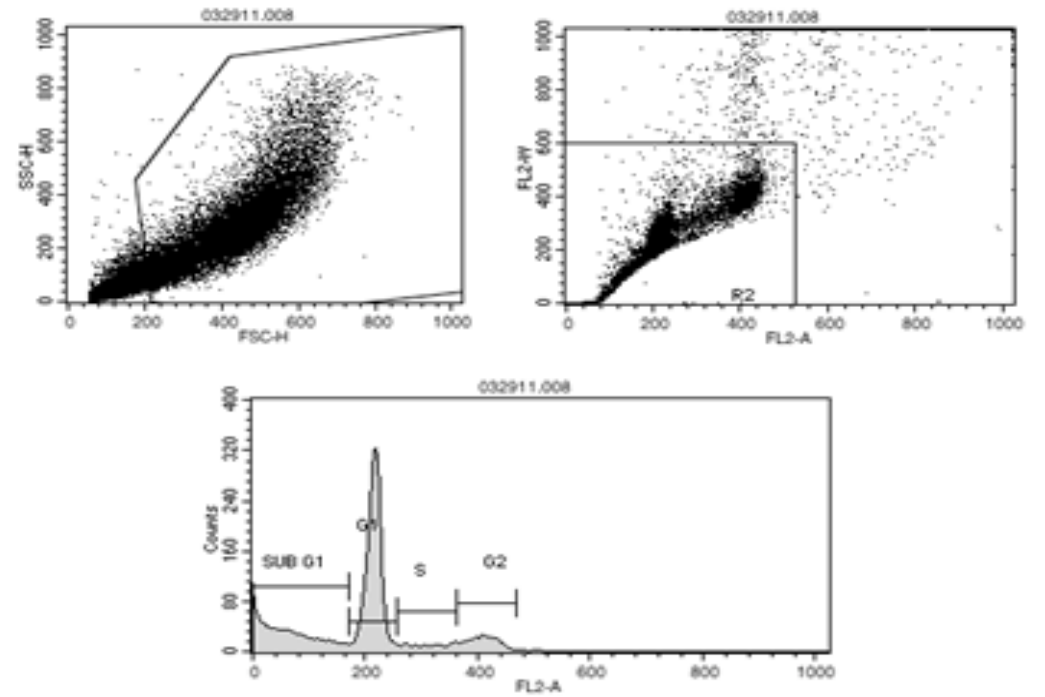

Supplement: S2 File — Note: the FACS plots are gated differently to those in Fig S1 in [1]. (PDF) [file pone.0346190.s002.pdf]

1C

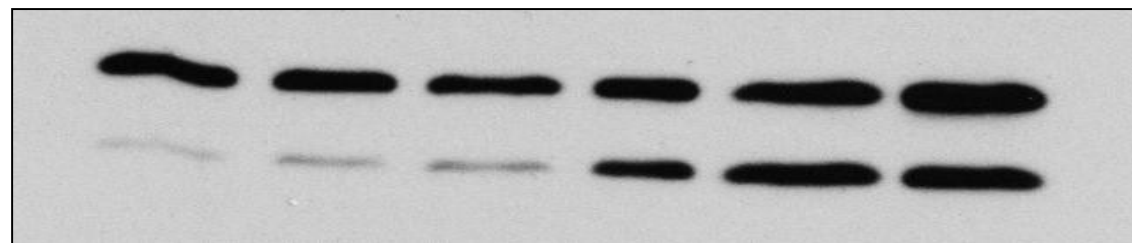

Con 6h 12h 16h 24h 36h

1D

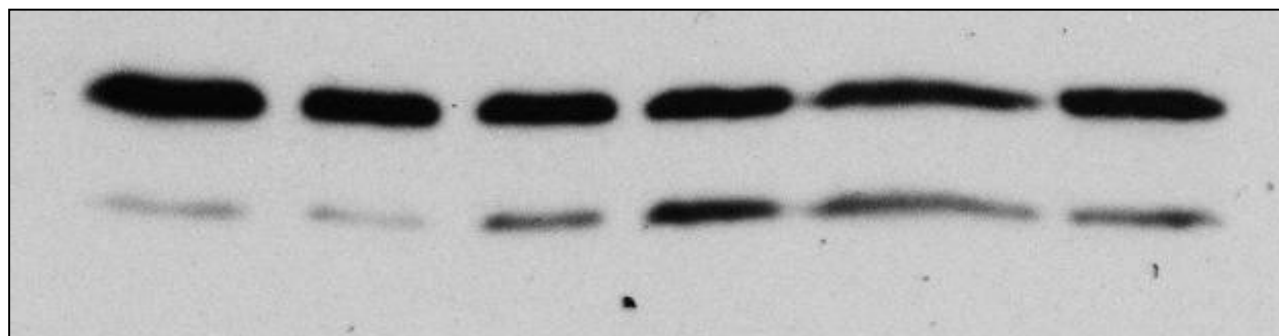

Con 6h 12h 16h 24h 36h

Supplement: S3 File — (PDF) [file pone.0346190.s003.pdf]
